# Technological Solutions and Main Indices for the Assessment of Newborns' Nutritive Sucking: A Review

**DOI:** 10.3390/s140100634

**Published:** 2014-01-02

**Authors:** Eleonora Tamilia, Fabrizio Taffoni, Domenico Formica, Luca Ricci, Emiliano Schena, Flavio Keller, Eugenio Guglielmelli

**Affiliations:** 1 Laboratory of Biomedical Robotics and Biomicrosystems, Center for Integrated Research, Università Campus Bio-Medico di Roma, Via Álvaro del Portillo, 21, Rome 00128, Italy; E-Mails: f.taffoni@unicampus.it (F.T.); d.formica@unicampus.it (D.F.); l.ricci@unicampus.it (L.R.); e.guglielmelli@unicampus.it (E.G.); 2 Unit of Measurements and Biomedical Instrumentation, Center for Integrated Research, Università Campus Bio-Medico di Roma, Via Álvaro del Portillo, 21, Rome 00128, Italy; E-Mail: e.schena@unicampus.it; 3 Laboratory of Developmental Neuroscience and Neural Plasticity, Center for Integrated Research, Università Campus Bio-Medico di Roma, Via Alvaro del Portillo, 28, Rome 00128, Italy; E-Mail: f.keller@unicampus.it

**Keywords:** nutritive sucking monitoring, oral feeding skills, smart objects, early neonatal assessment, clinical decision-making, at-home monitoring

## Abstract

Nutritive Sucking (NS) is a highly organized process that is essential for infants' feeding during the first six months of their life. It requires the complex coordination of sucking, swallowing and breathing. The infant's inability to perform a safe and successful oral feeding can be an early detector of immaturity of the Central Nervous System (CNS). Even though the importance of early sucking measures has been confirmed over the years, the need for standardized instrumental assessment tools still exists. Clinicians would benefit from specifically designed devices to assess oral feeding ability in their routine clinical monitoring and decision-making process. This work is a review of the main instrumental solutions developed to assess an infant's NS behavior, with a detailed survey of the main quantities and indices measured and/or estimated to characterize sucking behavior skills and their development. The adopted sensing measuring systems will be described, and their main advantages and weaknesses will be discussed, taking into account their application to clinical practice, or to at-home monitoring as post-discharge assessment tools. Finally, the study will highlight the most suitable sensing solutions and give some prompts for further research.

## 1. Introduction

A recent report of the World Health Organization (WHO) describes how the rate of preterm births all over the world is increasing [1]. This result is particularly interesting since prematurity is the leading cause of newborns' death and because premature newborns represent a copious and ever-increasing population at high risk for chronic diseases and neurodevelopmental problems. Feeding support is one of the possible strategies reported in [1] to reduce deaths among premature infants. Such intervention requires specifically designed tools to assess oral feeding ability, so as to provide clinicians with new devices that may be used for routine clinical monitoring and decision-making. Several studies [2–4] stress the importance of introducing oral feeding for preterm infants as early as the Neonatal Intensive Care Unit (NICU), highlighting the need of evidence-based clinical tools for the assessment of infants' oral feeding readiness. The need for reliable assessment of feeding ability is further highlighted by the American Academy of Pediatrics that included the attainment of independent oral feeding as an essential criterion for hospital discharge [5].

The acquisition of efficient Nutritive Sucking (NS) skills is a fundamental and challenging milestone for newborns. It is essential during the first six months of life and it requires the complex coordination of three different processes: sucking, swallowing and breathing. The development of such precocious motor skills depends on intact brainstem pathways and cranial nerves. Hence, the immaturity of the Central Nervous System (CNS) can affect oral motor functions [6] and/or cause the inability to successfully perform oral feeding [7–10]. NS is one of the most precocious goal-directed action evident in a newborn's movement repertoire, and it may provide an opportunity to investigate mechanisms of fine motor control in the neonate, as reported by Craig and Lee in [11]. For these reasons, sucking skills can provide valuable insights into the infant's neurological status and its future development [12–16]. Moreover, since sucking control involves similar oral motor structures to those required for coherent speech production, early sucking problems have also been suggested as predictors of significant delays in the emergence or development of speech-language skills [17,18].

The importance of early sucking monitoring has been confirmed over the years, and the need for reliable instruments for neonatal sucking assessment is stressed in several works [2,4,15,19], even though no standardized instrumental assessment tools exist as yet. NS assessment is in fact part of the clinical evaluation, but this is not carried out objectively. With few objective criteria for the assessment of its progress in the hospital, and no organized home follow-up care, poor feeding skills may go undetected for too long. Notwithstanding the ongoing development of tools for the assessment of NS, there is not a common approach to this issue, thus causing problems of variability of the measurements, as highlighted by several authors [9,15,19]. Such heterogeneity represents one of the causes of the discrepant findings reported in literature, and a major challenge in applying them to clinical practice, as reported by Slattery *et al.* in 2012 [15]. The use of standard pre-discharge assessment tools may foster the development of common quantitative criteria useful to assist clinicians in planning clinical interventions. Such devices, or a simplified version of them, might be adopted also for patients' follow-up, as remote monitoring of infants at home after discharge.

Section 2 provides a detailed survey of the main quantities and indices measured and/or estimated to characterize sucking behavior skills and their development. Section 3 presents the main characteristics of the technological sensing solutions adopted to measure the previously identified quantities and indices. Finally, we will discuss the main functional specifications required to a proper feeding assessment device, and the main advantages and weaknesses of the adopted sensing systems, taking into consideration the application to the clinical practice, or to at home monitoring as post-discharge assessment tools.

## 2. Nutritive Sucking Process

### 2.1. Preliminary Definitions

Sucking is one of the first oromotor behaviors to occur in the womb. There are two basic forms of sucking: Non-Nutritive sucking (NNS) when no nutrient is involved, and Nutritive Sucking (NS) when a nutrient such as milk is ingested from a bottle or breast. A nutritive suck is characterized by the rhythmic alternation of *Suction* (S), *i.e.*, creation of a negative Intraoral Pressure (IP) through the depression of jaw and tongue, and *Expression* (E), *i.e.*, the generation of positive Expression Pressure (EP) through the compression of the nipple between the tongue and the hard palate. This S/E alternation allows the infant to create the extraction pressure over the fluid, contained in a vessel, towards the oral cavity.

From birth throughout the first 6 months of life, infants obtain their primary food through NS. During this process, the infant must control oral sucking pressures to optimize the milk flow from the feeding vessel into the mouth, and to move the expressed milk to the back of the mouth, prior to being swallowed. The amount of milk entering the mouth dictates the swallow event, which in turn interrupts breathing. Hence, during NS, Sucking (Sk), Swallowing (Sw) and Breathing (B) are closely dependent on each other. This dependence represents another strong difference between NS and NNS: during NNS, the demands on swallowing are minimal (the infant has only to handle their own secretions), and respiration can operate independently. Safety in NS implies a proper coordination of Sk, Sw and B to avoid aspiration, as the anatomical pathways for air and nutrients share the same pharyngeal tract. During the Sw phase, airflow falls to zero, where it remains for an average duration of 530 ms, to be rapidly restored after this time. This period of flow cessation between functionally significant airflows is usually referred to as “swallow apnea” [20].

In full-term healthy infants, the NS process is characterized by a burst-pause sucking pattern where a burst consists of a series of suck events, occurring with a typical frequency of 1 Hz [21], separated by the following suck event through a pause of at least 2 s. This burst-pause pattern evolves during feeding in three stages: continuous, intermittent and paused [22]. At the beginning of a feeding period, infants suck vigorously and continuously with a stable rhythm and long bursts (continuous sucking phase). This phase is generally followed by an intermittent phase in which sucks are less vigorous, bursts are shorter and pauses are longer (intermittent sucking phase). The final paused phase is characterized by weak sucks and very short sporadic bursts.

**Figure 1.**
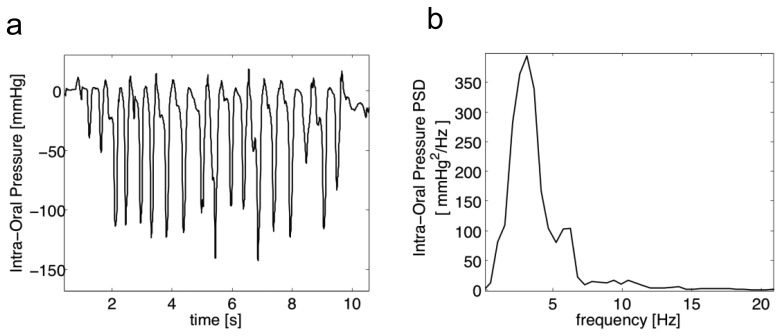
(**a**) IP of a 1-week healthy subject during bottle feeding; (**b**) Power Spectral Density (PSD) of intraoral pressure during NS (adapted from [23] with permission).

Figure 1 reports a typical 10 s pressure burst: experimental data, acquired on healthy subjects and reported in [23], showed that intraoral pressure is in the range of [−140,+15] mmHg. The bandwidth of the pressure signal was estimated, calculating its Power Spectral Density (PSD) by means of the Welch overlapped segmented average: it may be considered well below 20 Hz. Moreover, in a coordinated cycle of NS, the 1:1:1 relational pattern among sucking (S/E), swallowing and breathing is expected, and creates a rhythmic unit where breaths seem uninterrupted (no asphyxia or choking signs) [22].

### 2.2. Nutritive Sucking Behavior Monitoring and Assessment: Measured Quantities and Principal Sucking Parameters

The ability to nutritively suck is not always completely mature in infants at birth and may require time to develop or to mature. For immature infants, the developmental complexity of the feeding process can cause a series of difficulties associated with the initiation and progression of feeding from a bottle, which is the most frequent indicator of the discharge readiness adopted by healthcare personnel [24]. Bottle feeding indeed has been widely investigated because of this reason, and because it allows standardization or control of some feeding characteristics across infants (e.g., liquid composition, nipple hole size, and hydrostatic pressure of milk) [25]. For the same reasons this review work is focused on the tools adopted for the assessment of infants' NS skills during bottle feeding. The adoption of instrumental measures for this early assessment (in opposition to non-instrumental observational methods) is increasing, because of the growing interest in standardized, reliable, and valid measures of oral sensorimotor function in infancy [9]. Indeed, such instrumental measures of early oral feeding ability have been reported to be more sensitive and specific to predict later neurodevelopment outcomes, compared to non-instrumental observational tools [15], whose psychometric properties are still debated [26].

Literature reports this instrumental assessment of NS behavior and of its development through the analysis of a wide variety of indices that could be extracted from the measurements. The identification of the most significant indices may be important to lead future research to focus on their investigation and on the establishment of normative data for the identification of deviations from the norm. We have focused on providing a survey of the principal indices used for the assessment of NS behavior, as well as of the quantities measured to extract them. Table 1 reports the most significant indices adopted for the instrumental assessment of infants' NS behavior during bottle feeding. The indices have been grouped in three main categories according to the final objective of the assessment: (i) to evaluate the level of maturation of oral feeding skills in preterm infants [25,27–36]; (ii) to evaluate or characterize the level of maturation of oral feeding skills in term infants [37–43]; and (iii) to make an early detection of later neurodevelopmental outcomes [13,14,36,44]. Table 2 lists the different physical quantities that have been measured to monitor the NS process and from which the evaluation indices have been extracted. Both tables are organized in order to separate the different components of the NS process, *i.e.*, sucking, swallowing, breathing, and nutrient consumption.

For the assessment of preterm infants' maturation in terms of sucking skills during bottle feeding, several indices have been adopted. The organization into bursts and the establishment of a stable temporal pattern are important developmental steps in the maturation process of the sucking component [30]. Some descriptive parameters represent important indices for the evaluation of this maturation, *i.e.*, the number of sucks per burst and the percentage of sucks in bursts. Moreover, the number of sucks composing the first burst has turned out to be a useful indicator of the feeding outcome [31]. In addition to these descriptive parameters, several temporal parameters appear to be consistent indicators of preterm infants' maturation, such as: sucking frequency (sucks per min), burst duration, inter-burst width, inter-suck width, and an index of rhythmic stability referred to as Coefficient of Variation of the Sucking process (COV_Sk_). This index is adopted by several studies to assess the maturational patterns in terms of rhythmicity [29,30,33], and it is defined as follows:

(1) $${\text{COV}}_{X}:=\frac{SD(I)}{\text{mean}(I)}$$

where *SD* is the standard deviation, and *I* represents the time interval between two consecutive events of the considered *X* process (e.g., the interval between consecutive sucks). All these indices can be calculated measuring any quantity that allows the identification and the temporal characterization of sucking events, without distinction between suction and expression components. For example, these parameters can be estimated even through measures of intranipple pressure or chin movements, as in [30,31]. On the other hand, the specific measure of the suction component (IP) is very frequent for the assessment of NS skills. This allows to estimate all the indices already mentioned, as well as the maximum suction amplitude the infant is able to generate (IP amplitude), which is reported as an indicator of the preterm infant's suction maturation [25,27–29,32]. However, the maturational process of preterm infants' oral-motor skills has been proven to be characterized by some developmental stages defined according to indices of both expression and suction components [28]. Preterm infants develop and establish first the expression component, then suction, and finally the S/E rhythmic alternation. Hence, measures of both sucking pressures (IP/EP) are needed to estimate some significant indicators of this maturational progress [28,29]: S and E rhythmicity, the S:E ratio, the time interval between S and E (S-E interval), IP and EP amplitude.

The maturational level of sucking skills in term infants appear to be completely assessable through a set of descriptive and temporal indices, that do not require the measurement of both sucking pressures [37–42]. Almost all of these indices have been already mentioned. An additional one is introduced in [37] to quantify the sucking variability, through a measure of the suck-to-suck fluctuation in amplitude. The authors refer to it as *inconsistency* index and define it as the SD of the ratios of amplitudes of successive sucks within bursts. Moreover, an index of sucking *intensity* is defined as the mean maximum sucking pressure divided by the mean suck duration, and appears to be correlated with the efficiency of the sucking pattern [41].

However, as Table 1 reported, some significant indices for the assessment of the oral feeding maturation also concern other components of the NS process. Immature NS does not only reflect sucking ability, but also the coordination of suck with swallowing and respiration. Among the principal indices adopted for the evaluation of these coordination skills in preterm infants, there are the coefficients of variation calculated from the breath-breath and swallow-swallow intervals (COV_B_ and COV_Sw_), that allow the analysis of the feeding-related respiratory and swallowing rhythms [33]. Another significant index is the percentage of apneic swallows, *i.e.*, the number of series of at least three swallows not associated with breathing events, divided by total swallows. This index appears to be a clear indicator of maturation in bottle-fed preterm infants, as reported in [43], stressing the importance of the ventilatory control during feeding. However, the maturation of this aspect cannot be complete at term gestation, hence this index of deglutition apnea represents an indicator of term infants assessment as well. Moreover, a safe coordination between Sw and B is reported as an important developmental achievement for preterm immature infants, and it is usually assessed as the percent occurrence of a specific Sw-B interface (e.g., Inspiration-Swallow-Expiration, I-S-E) [25,29,33]. This index is also an important indicator for the assessment of the term infant's feeding pattern, along with the sucking-breathing (Sk-B) interface and the Sk:Sw:B ratio [41].

All these parameters to evaluate the preterm infants' ability to establish a mature coordination between sucking, swallowing and breathing, can be estimated from different measures of swallowing and breathing (see Table 2), which allow the detection of the events (swallows, inspirations, expirations).

For both preterm and term infants, oral feeding performance is usually assessed through indices of sucking efficiency (usually defined as the nutrient volume per suck) and the rate of nutrient intake (intake volume divided by feeding duration), calculated using all the different measures of nutrient consumption adopted and reported in Table 2. An alternative definition of the sucking efficiency that has been adopted is the average milk intake per suck divided by average effect (pressure · duration) per suck [41]. The bolus size (volume per swallow) is another index of nutrient consumption that allows to assess feeding performance in relation to the swallowing pattern.

Nutritive sucking has also been considered as an early motor marker for the prediction of later neurodevelopmental outcomes in infants [15]. Some already mentioned sucking indices (Sk frequency, number of sucks per burst, IP amplitude) have turned out to be predictors of later neurological outcomes [13]. Moreover, with measures of both suction and expression components (IP and EP), the newborn's sucking pattern has been classified according to the rhythmicity and amplitude of both components, inferring prediction of later neurological development [14]. S and E have also been assessed, through measurements of throat and jaw movements [44] (see Section 3 for additional details). The eye-jaw and eye-throat distances have demonstrated to be useful to identify the differences in feeding performance between healthy infants and infants with neurological disorders. Nutrient consumption is another important factor whose monitoring can allow the estimation of significant indices with predictive value. The newborn's feeding behavior, assessed through the milk intake rate (mL/min), has demonstrated to be correlated with future neurodevelopment assessment [36].

No measurements of the other components (breathing, swallowing) of the nutritive sucking process have been carried out to this aim. Such predictive potential of sucking assessment was also confirmed by other authors [6,45,46] whose studies are not reported in this work since they adopted non-instrumental tools for the assessment.

The importance of the instrumental monitoring of NS has also been demonstrated in the case of neurodisabled infants with Down's syndrome: the use of sucking pressure waveforms (IP and EP measures) can be helpful in the examination of the development of sucking behavior, intraoral movements and therapeutic effects [35]. Moreover, problems with sucking and swallowing can be observed in children with cerebral palsy (CP) within the first 12 months of life, which often precede the diagnosis [6,47]. These observations emphasize the importance of monitoring feeding behavior, preferably at home, and taking a careful feeding history.

## 3. Sucking Behavior Monitoring and Assessment: Technological Solutions and Methods Adopted in Research Studies

In the previous section we have reported the complex set of indices and quantities, used to objectively assess infant oral feeding. The measurement of such a heterogeneous set of indices requires several technological solutions that can be grouped into three categories: (i) measuring systems to monitor sucking process (Table 3); (ii) measuring systems to monitor swallowing and breathing process (Table 4), and (iii) measuring systems to monitor nutrient consumption (Table 5). Swallowing and breathing are considered together, because several authors [29,32,33,35,41] demonstrated the oral feeding performance in preterm infants to depend mainly on their coordination.

### 3.1. Measuring Systems to Monitor Sucking Process

The literature suggests several methods to monitor the Sk process. Such methods rely on Pressure Transducers (PTs), optical motion capture systems, and resistive strain gauges to monitor EP and IP, chin, throat, and jaw movements (see Table 3).

**Figure 2.**
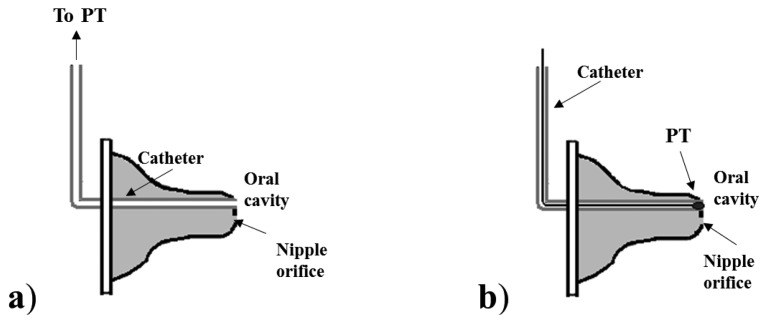
Schematic diagrams of a nipple designed for nutrient delivery and IP monitoring: (**a**) PT connected to an end of a catheter whose other end is connected to the oral cavity; (**b**) PT inserted in the catheter and directly flushing in the oral cavity.

PTs are usually adopted to measure both IP and EP. In particular, the measurement of IP is always performed using PTs, but adopting two different nutrient delivery systems. In the first one, a common bottle nipple is used (Figure 2): a catheter is applied to the tip of the nipple for IP measurement, while the nutrient flows from the lumen of nipple to the infant's mouth through the orifice normally present on the nipple tip. This configuration has been adopted in several studies, which have used two different sensing solutions for IP measurement, depending on the position of the transducer and on its type, as illustrated in Figure 2: a small pressure catheter (e.g., Millar Mikro-Tip SPR-524) is used and directly placed at the nipple tip [28,29,32,34,48], or a semiconductor PT is connected to the end of a catheter whose tip is placed into the oral cavity [10,14,16,25,35,41,44,49–51].

**Figure 3.**
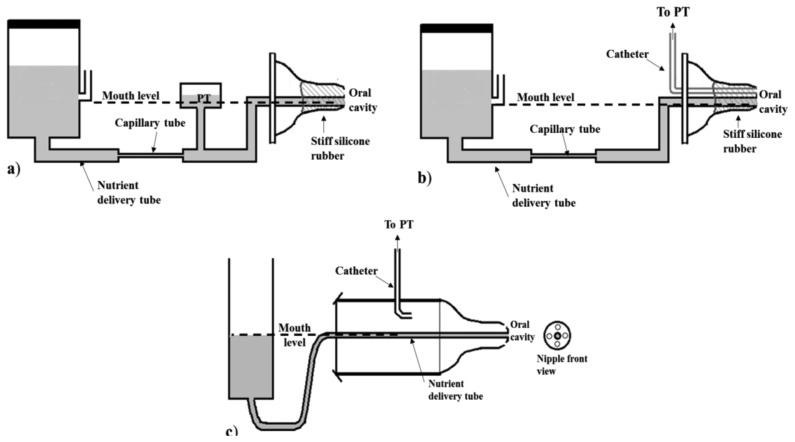
Schematic diagrams of feeding apparatuses designed for nutrient delivery and IP monitoring: (**a**–**b**) configurations including a capillary tube, where a PT measures pressure changes between the nipple and the capillary tube (**a**), or in the oral cavity through a second catheter inserted into the nipple (**b**); (**c**) a PT measures the pressure changes in a chamber directly communicating with the oral cavity, and the nutrient is delivered from an open reservoir through a catheter into the mouth.

Some studies [16,50,51] which use the first configuration specify that the catheter used is filled with fluid for a more robust pressure measurement, less sensitive to artifacts; in the others using the same configuration, it is not specified. The nipple can be also standardized and calibrated so that it responds to a certain differential pressure (difference between intranipple and intraoral pressure) with a known and acceptable milk flow rate, as in [14,25,35,57]. In the second configuration (see Figure 3), the nipple is modified to embed a tube for nutrient delivery within the nipple tip, and a second tube is connected to a PT to measure IP pressure. The properties of the nipple in this case do not completely resemble an ordinary nipple: it is not filled with fluid, so expression movements cannot influence the nutrient's release. This configuration implies that nutrient flows only when the infant develops an appropriate IP.

One of the earliest works studying Sk [39] describes the use of a capillary tube as flow meter. The system adopted in this study is composed of a stoppered burette connected to a capillary tube and then to a nipple. To guarantee a constant delivery pressure equal to the atmospheric one, an opening is placed to a side arm of the burette and always kept at the same height as the nipple. The flow-limiting capillary tube allows to regulate the flow of nutrient introducing a known linear relation between IP and flow throughout the range of infant sucking pressures (considered as 0 to −300 mmHg). Since such arrangement may be considered a closed hydraulic system, any increase or decrease of pressure applied to the nipple is transmitted to every part of the connected system. In particular, since the capillary can be considered as a concentrated resistance, it is possible to measure the main pressure drop along it, and a pressure equal to the desired IP in the part after it.

Figure 3a shows the described configuration where the PT is placed between the capillary and the nipple. A similar capillary system has also been adopted in later studies [8,13,27] where the PT is specified to be connected to the oral cavity by means of a second catheter inserted within the nipple, as Figure 3b illustrates. The nipple is stiffened with the use of a silicone rubber, in order to prevent nutrient delivery through expression movements, in both systems. With this arrangement, the nutrient flow rate through the tube can be calibrated, so that a certain intraoral pressure provides a known flow rate, and the consumption is proportional to the pressure-time integral. This calibration is allowed thanks to a proper configuration of the feeding system which eliminates two influencing factors: the hydrostatic pressure caused by the height of the level of nutrient over the infant's mouth, and the gradual vacuum increase inside a sealed nutrient reservoir as the milk flows out. Particular attention has to be paid in order to limit the effects of these two factors, which influence the net pressure forcing the liquid into the mouth thus hampering the feeding performance [28]. In Figure 3c another feeding apparatus adopting such expedients is shown. An opened reservoir is used to avoid vacuum creation, and the level of the nutrient is constantly maintained at the level of the catheter tip to eliminate any hydrostatic pressure. This measuring system, adopted in [54], embeds two catheters: one for nutrient delivery into the oral cavity, and a second one ending into the same chamber as the nipple which is in direct communication with the oral cavity thanks to some holes in the nipple tip. This nutrient delivery system allows the establishment of a linear flow rate in the catheter over a range of 1 to 100 cm H_2_0 of infant sucking pressures.

**Figure 4.**
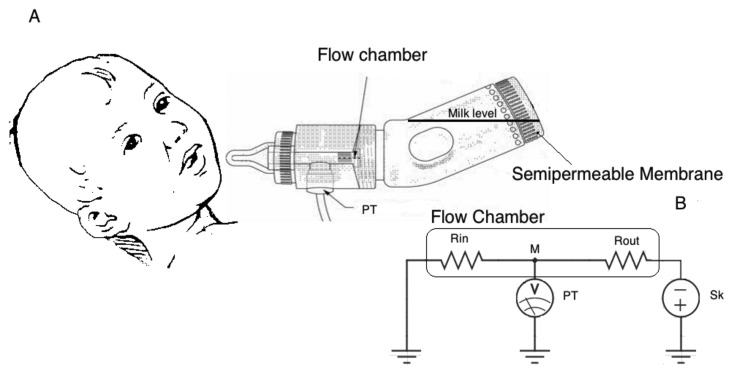
(**a**) Portable sensing feeding apparatus for IP monitoring: the nutrient is released into the infant's mouth through a tube connected to a common nutrient reservoir. The tube presents an orifice restriction at the beginning and a PT for IP measurement before the nipple; (**b**) Equivalent electronic circuit of the apparatus.

Lang and colleagues [37] developed a solution, very similar to an ordinary feeding bottle, embedding a nutrient delivery tube which enables a higher level of portability (see Figure 4a). They use a modified commercial bottle (VentAire feeding bottle, produced by Platex) where a flow chamber is inserted between the milk reservoir and the outlet. The chamber has an inlet flow restriction orifice and an anti-backflow valve. The inlet chamber diameter is very small with respect to the outlet, offering a higher resistance to milk flow. The pressure inside the milk reservoir is maintained at the atmospheric value thanks to a gas permeable, fluid impermeable membrane. The shape of the bottle reduces the effect of the hydrostatic pressure allowing for an easy adjustment of the level of milk to that of the infant's mouth. Such system allows monitoring the suction pressure by measuring the pressure changes inside the chamber. The system may be modeled by the equivalent electronic circuit reported in Figure 4b. The inlet and outlet diameter are represented by two electrical resistances, R_IN_ and R_OUT_ respectively; the PT measuring the pressure (voltage) inside the flow chamber with respect to the atmospheric pressure (GND) is modeled by a voltmeter connected to the measuring node M; finally, sucking pressure is represented by a voltage generator (Sk). The voltage measured at node M will be:

(2) $$V_{M}=\frac{R_{in}}{R_{in}+R_{\text{out}}}V_{Sk}≅V_{Sk}$$

since R_in_ >> R_out_ (due to the geometry), V_M_ may be reasonably assumed as equal to V_Sk_.

**Figure 5.**
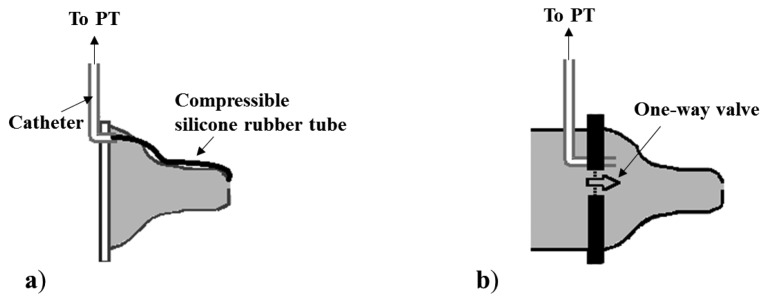
Sensing solutions for EP measurement: (**a**) a PT measures EP by means of the compression of a silicone tube inserted into the catheter connected to the transducer; (**b**) a PT is connected via a catheter to the lumen of the nipple (continuously filled with liquid); a one-way valve between the nutrient chamber and the nipple is applied.

**Figure 6.**
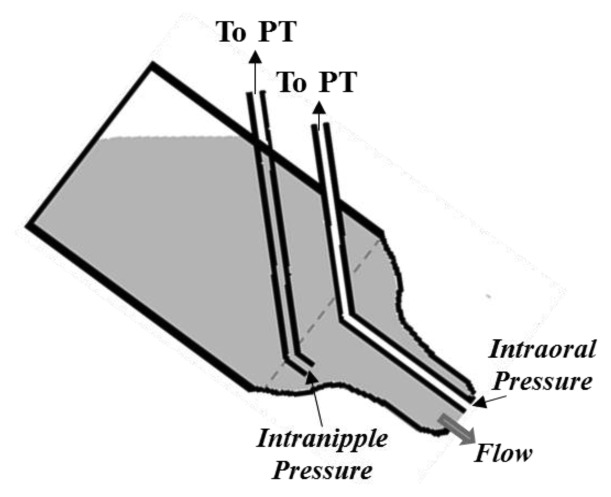
Sensing solutions for the measurement of the net pressure forcing the nutrient out of the nipple chamber. Two PTs are adopted to measure intranipple and intraoral pressure, and calculate the pressure gradient causing the nutrient to flow out.

For the EP measurement, a rubber silicon tube can be placed on the outer surface of the nipple of a feeding bottle [27,29,32,34,48] (see Figure 5a). One end of the catheter (the extremity inside the mouth) is closed, while the other end is connected to a PT by means of a polyethylene catheter. This measuring system presents a limitation due to the rapid reaching of a plateau corresponding to the catheter's full compression. Otherwise, a PT can be connected to the lumen of the nipple by a silicone catheter to measure EP. In particular, EP is measured using this configuration and adding a one-way valve between the nipple chamber and the nutrient reservoir [14,35,49] (see Figure 5b). Such a valve allows to isolate the interior of the nipple from the milk reservoir during the expression phase, in order to ensure that the nipple be always full. The same configuration without the valve allows monitoring the intra-nipple pressure changes due to the sucking events with no S/E distinction [30,33,40,52]. Moreover, McGowan *et al.* [51] estimate the net pressure forcing the nutrient out of the nipple as the difference between intra-nipple pressure and negative intraoral pressure measured outside the nipple (see Figure 6).

**Figure 7.**
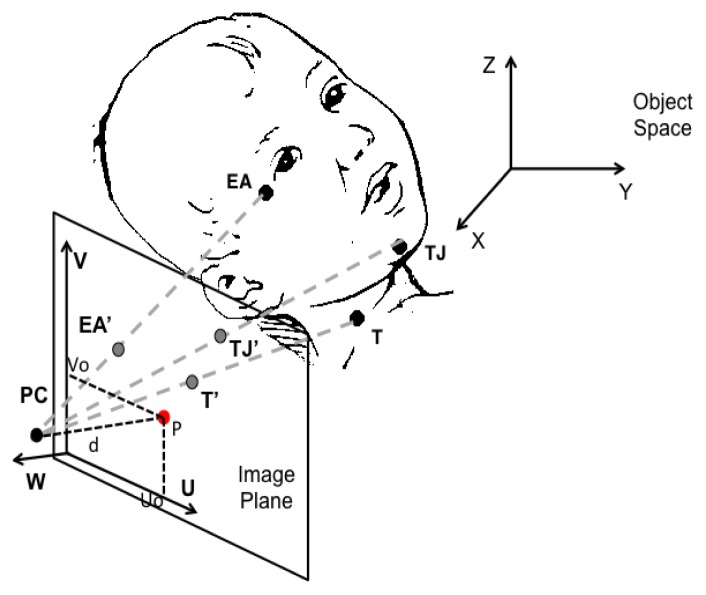
Position of the marker on the throat region, for DLT method application, is determined by first locating three facial markers: the external eye angle (A), the tip of the jaw (B) and the throat region (C).

The two different sucking components can also be monitored through the measurement of throat and jaw movements. Such movements are assessed adopting two different technological solutions in [10,44]. One includes the use of a strain-gauge transducer attached between the infant's forehead and the chin [10], to measure jaw movements associated with mouthing movements. In [44] authors use a 1 meter distant camera placed at 90° with respect to the front of the baby's face. Three markers are placed on the infant' s face (see Figure 7): one on the lateral Eye Angle (EA), one on the Tip of the Jaw (TJ), and the last one on the Throat (T). To estimate the distance of the marker in the object plane, the Direct Linear Transformation (DLT) method is used. Such method allows the definition of a linear transformation between the object space and the image-plane reference frame. Considering a point *T* in the object space (*T* = [*x*,*y*,*z*]*^T^*), it is mapped onto the image plane as *T′* and expressed in the image reference frame as *T′* = [*u,ν*,0]*^T^*. We can write:

(3) $$\left[\begin{array}{c} u-u_{0}\\ \nu -\nu_{0}\\ -d \end{array}\right]=c\left[\begin{array}{ccc} r_{11} & r_{12} & r_{12}\\ r_{21} & r_{22} & r_{23}\\ r_{31} & r_{32} & r_{33} \end{array}\right]\left[\begin{array}{c} x-x_{0}\\ y-y_{0}\\ z-z_{0} \end{array}\right]$$

where *x*_0_, *y*_0_, *z*_0_ and *u*_0_, *ν*_0_, *d*_0_ represent the coordinates of the Projection Center (PC) respectively in the object reference frame and in the image reference frame; *c* represents a scale factor and the matrix *R* a transformation matrix which allows the projection from one space to the other one. The elements of this matrix are estimated thanks to a preliminary calibration procedure after which the camera should not be moved. Authors simultaneously recorded IP, EP and two anatomical distances, *i.e.*, the eye-throat and the eye-jaw distance, and proved their correlation with suction and expression pressures respectively.

In other works [31,53], mercury-in-rubber strain gauges are used to monitor chin movements. Such sensors are connected to a plethysmograph that detects changes in electrical resistance of the gages as they are stretched with sucking activity. Strain gage is kept under tension during measurements, stretching it by at least 10% to 20% beyond its resting length before application. Such set up demonstrates reliability in sucking monitoring, even distinguishing chewing on the nipple and other non-sucking activity from true sucking [58].

### 3.2. Measuring Systems to Monitor Swallowing and Breathing Processes

The evaluation indices on swallowing and breathing concern rhythmicity and their interface, rather than their “extent” (see Table 1). Different solutions in terms of complexity have been adopted to monitor these two processes.

Sw events are often detected by monitoring the pharyngeal pressure with a PT connected to a tube, transnasally inserted as far as the pharynx [25,30,33,40,50,52]. Otherwise, a PT connected to a small drum, placed on the hyoid region of the infant's neck, is used to monitor hyoid bone movements related to swallowing [29,32,48]: the upward movement of this bone, caused by Sw, results into a biphasic pressure wave in the drum, and the peak pressure deflection is generally used as marker of Sw. Other authors [10,41,49] use microphones placed on the infant's throat to record swallow sounds. Such microphones need to be small enough to be effectively applied to infant and their bandwidth should cover a range from 100 Hz to 8 kHz [59]. Moreover, they require to be shielded from external noise and to filter out some possible interference, such as e.g., babbling. The acoustic technique to record swallowing in premature infants is widely investigated also in [60,61].

**Figure 8.**
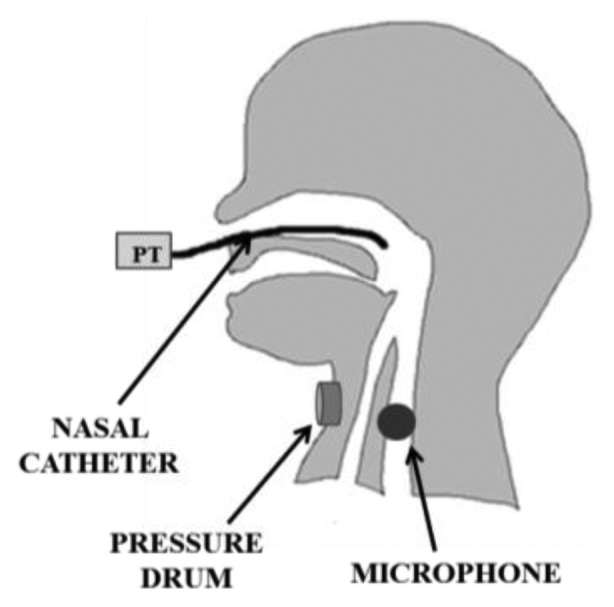
Different devices used for swallowing monitoring: PT connected to a transnasal catheter; pressure drum applied on the hyoid bone; microphone applied on the throat.

**Figure 9.**
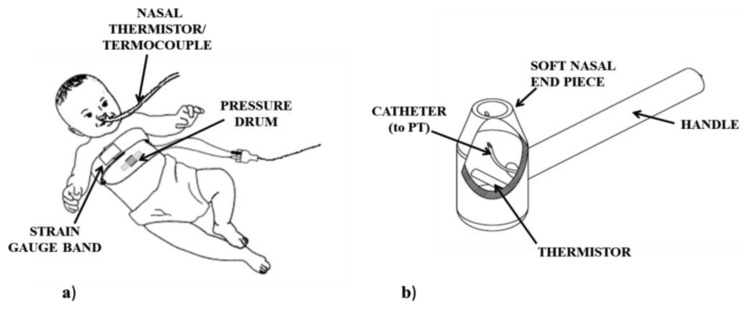
Devices used for breathing monitoring. (**a**) Nasal thermistor or thermocouple applied below the nostrils for nasal airflow measurement; pressure drum or strain gauge band on the chest for respiratory movements measurement; (**b**) Rigid tool applied into the nostrils: the thermistor and the PT are used respectively to assess air flow and its *versus*.

Breathing process is monitored measuring nasal airflow and/or respiratory movements. All the different sensing solutions for respiratory monitoring during feeding in infants are shown in Figure 8. Nasal thermistors or thermocouples below the nostrils are used to measure air flow. However, they are not sufficient to distinguish between inspiration and expiration. To address this issue, one possibility is represented by the use of additional sensors. To clearly identify flow direction, a PT connected to the nostrils by a soft catheter can be used [10]. Catheter and thermistor are embedded into a rigid tool (see Figure 9b), which is kept in the infant's nostrils during feeding, capable of recording very low airflows (discrimination threshold less than 0.5 L/min), without adding any significant resistance to the flow. Another solution that allows measuring both the airflow and its direction is the use of a miniaturized pneumotachograph connected to a pressure transducer, placed in a nostril. Such nasal flowmeter has turned out to be suitable for preterm infants [43], because of its low dead space (less than 0.11 mL), low resistance (0.1 mm H_2_O/mL·s), light weight (0.2 g) and compact design.

Airflow monitoring is also widely performed by thermistors because of their rapid response to flow changes [10,25,30,40,49]; however, they are prone to artifact caused by temperature equilibration when airflow stops [62]. To avoid such problem, many authors monitor breathing by measuring thoracic movements (see Figure 9a). Such measures pertain to changes in lung inflation measured by chest and abdominal movements, and enable to determine the precise timing of the end of inspiration and expiration, not allowing for quantitative measures such as tidal volume or minute ventilation. Mercury-in-rubber or piezo-resistive strain gauges (respiratory bands) have been used to measure chest movements [25,30,33,41,50], as well as PTs, connected to a drum taped at the thoraco-abdominal junction [29,32].

### 3.3. Measuring Systems to Monitor Nutrient Consumption

Nutrient consumption is often estimated measuring the residual nutrient volume at the end of the feeding session (total consumption) or at variable time intervals. This measure is frequently performed observing the liquid level in a graduated reservoir [28,32,39] or using a balance [29,48]. Many authors do not even mention this measure, despite reporting consideration about the total ingested nutrient volume [25,40,41]. Measurements of the nutrient consumption at very close intervals of time have also been adopted [23,50,56]. The volume of delivered milk can be estimated measuring the changes of the air pressure inside a closed bottle (vacuum built-up) by means of a PT, while the liquid flows out, as reported in [23]. A PT can be also used to measure the hydrostatic pressure of the remaining liquid column in a cylindrical reservoir in order to estimate the residual volume of liquid, as presented in [56]. In this work, the PT was connected to an air-filled catheter ending at the base of an inverted bottle where the liquid column laid. Such sensing system also included the presence of an accelerometer to estimate the bottle tilt and correct its influence on the hydrostatic pressure. The same principle was also adopted by Al-Sayed [50], who measured the hydrostatic pressure of the residual volume of milk, but in a more controlled situation where the reservoir was fixed and could not be tilted. Such approach enabled to measure the residual nutrient volume in the bottle at variable temporal intervals, when there was no sucking activity. Measures of the flow of nutrient have been performed using particular flow meters [10,54,55]. In [55], the authors use an ultrasonic flow transducer to measure the liquid flow between the feeding bottle and the tip of the feeding nipple. In the other two studies the milk flow is estimated from the measurements of airflow entering the reservoir to fill the void left by the milk, using a pneumotachometer [54], or a thermistor [10]. In addition to all these methods, many studies, as already described in Section 3.1, estimated the milk flow rate using a calibration of the nutrient delivery system.

## 4. Discussion

Oral feeding is a complex process requiring a mature sucking ability and an especially mature coordination of sucking with breathing and swallowing. The proposed overview of scientific literature about this topic highlighted the absence of a unique technological sensing solution to assess such skills. Oral feeding behavior is assessed in literature, monitoring sucking, breathing, swallowing, and nutrient consumption, through a wide set of quantities and indices. Such monitoring has resulted potentially useful for the assessment of oral feeding pattern maturation in preterm infants, in term infants, and as prognostic tool for predicting later neurodevelopmental outcomes.

To study preterm infants' feeding behavior, intraoral and expression pressures are two fundamental quantities. In particular, S/E coordination and rhythmicity are principally investigated and are significant to characterize and assess the development of sucking skills and, possibly, their immaturity. Besides, the importance of these two components has also been confirmed in the case neurodisabled infants, confirming the importance of S/E monitoring for the assessment of immature sucking patterns. On the contrary, sucking skills of term infants can be assessed without distinction between IP and EP measures. Regarding the prognostic value of infants' oral feeding skills, it seems that the assessment of the only sucking pattern is sufficient to predict later neurodevelopmental outcomes. However, the reported studies [13,14,43], dealing with the issue, are rather recent and encourage further research focused on breathing and swallowing processes as well [36].

Both for preterm, and term infants, Sw-B coordination represent a challenging milestone to attain in development of feeding skills. Sw-B rhythmicity and their integration into the sucking process is fundamental, highlighting the importance of measuring systems for the detection of Sw and B events and their temporal pattern, rather than for their quantitative characterization.

Volume consumption was monitored in most of the studies, and feeding efficiency, as well as feeding rate and bolus size, appear as important indices to evaluate the changing feeding performances in both preterm and term infants.

Such heterogeneous set of quantities and indices has been assessed with different technological solutions. Due to the differences related to the application field (preterm assessment; term assessment; prognostic tool), each solution should be carefully assessed according to the specific application requirements, which should always aim at a balanced compromise between reliability, invasiveness and practicability.

The distinction between suction and expression components of sucking seems to be a characteristic specific of the assessment of preterm infants. PTs are the most commonly adopted sensing solution for the measurement of intraoral and expression pressures. However, the simultaneous monitoring of both sucking components imposes special constraints. In particular, the measuring configuration including a tube for nutrient delivery (Figures 3 and 4) cannot be adopted, because it removes the contribution of the expression component on the extraction of nutrient and it drives infants to modify their sucking response [63] forcing a higher IP. This solution can be adopted if the only goal is to investigate suction ability, since it forces the newborn to rely on it. Moreover, the specific application to preterm infants imposes additional constraints, due to the compromised motor system of this population. The presence of an additional resistance to the nutrient flow, caused by the tube restriction (see configuration in Figure 3a,b and Figure 4), makes such measuring solutions unsuitable when applied to preterm infants: additional resistance implies additional sucking efforts to extract the same amount of nutrient from the bottle. Furthermore, the acceptability of the feeding apparatus is essential for every application. A nutrient delivery tube within the nipple tip may compromise the nipple's mouth feel, even for full-term and/or healthy infants accustomed to feeding from standard nipples on commercial bottles: they often refuse anything other than their usual nipple style [57]. For such reason, the relative simplicity of the common orifice nipples adapted for IP monitoring, as shown in Figure 2, is more advantageous. With such a configuration, infants' expression movements can alter flow rate through the nipple, and so the simultaneous measurement of EP, which is fundamental for preterm infants' assessment, makes sense. Both sensing solutions, illustrated in Figure 2, are applicable on every feeding apparatus and nipple, not requiring a particular design: they can be embedded in both a clinical and a portable domestic assessment tool. Besides, they can be also adopted for IP monitoring during breastfeeding [42,64]. The sensing solution where the pressure waveform is directed to the PT by means of a catheter (Figure 2a and Figure 3), should always adopt fluid-filled catheters (free of air bubbles), because air-filled lines do not respond to rapid pressure changes and underestimate peak negative pressures. However, the PT directly placed into the infants' mouth (see Figure 2b) is more advantageous for several aspects (higher accuracy, no time delay, no motion artifacts), also avoiding the need for a fluid-filled system (higher easiness of use), but it implies higher costs.

Regarding the measurement of EP using PTs, both reported methods (Figure 5) appear to be suitable for clinical and domestic use, as they can be incorporated in a standard feeding apparatus. However, the one measuring EP from the nipple lumen is suggested since the other one presents limitations due to a plateau in the system response corresponding to a full compression of the catheter.

Optical motion capture systems may be also considered for E and S monitoring through jaw and throat movements. The advantage of such monitoring approach is its complete non-invasiveness: any feeding apparatus can be adopted (depending on clinicians' or parents' decision), as no sensing elements are required (it can be even adopted for breastfeeding monitoring [64]). Notwithstanding such advantage, its practicability is very low, as it would require specialized personnel, a structured environment, precise calibration procedure and it would be time consuming. These reasons make this kind of monitoring system not easily practicable both for clinical and domestic post-discharge application. Moreover, mouthing movements (jaw movements) are not directly linked to the effective nutrient expression, as infants could have an ineffective seal around the teat, which would prevent them from feeding properly.

If no S/E distinction is necessary, sucking events can be monitored. The intranipple pressure can be easily recorded adopting a non-invasive and practical sensing solution, even embeddable in a common feeding apparatus. Sucking movements can also be recorded, adopting mercury-in-rubber strain-gauges on the infant's chin. The advantages of this latter solution is the fact that it does not require any special sensors to be applied on the feeding apparatus, which can consequently be freely selected by the user (it could be also used for breastfeeding monitoring). However, it is moderately invasive as the sensing element has to be placed on the infant's face. This may produce additional stress to the preterm newborn who often shows hypersensitivity of the facial area due to frequent necessary aversive oral and/or nasal procedures [29].

Two additional aspects should be taken into consideration in the definition of the main requirements of a standardized assessment tool for feeding: the hydrostatic pressure and the gradual build-up in negative pressure inside the bottle. In most feeding apparatuses, used to enable measures of feeding behavior, particular expedients were required to avoid these two factors, which might hamper the feeding performance of immature infants [28], so as to permit the standardization of feeding across infants and the generalization of results. The adopted expedients often imply solutions showing low practicability and requiring structured environment, not suited for post-discharge use (see the schematic representation in Figure 3). More efforts are required to design and develop measuring feeding tools, which can be easy-to-use also in post-discharge usual environment. While the vacuum problem can be easily avoided using a commercial vented bottle, as in [37] (Figure 4) or in [23], the hydrostatic pressure might also represent an important parameter to record in the case of daily home monitoring of sucking behavior, when the infant has to face this factor while bottle-feeding. Some described apparatus [51,56] can be adoptable for such application: they report sensing solutions based on PTs measuring the pressure at the base of the nutrient column. Moreover, these factors suggest that another important aspect in the design of a standardized feeding assessment tool, is its shape [65], as it can determine the extent of the influence of hydrostatic pressure on feeding (see Figure 4).

The Sw-B coordination is a challenging milestone in the development of oral feeding skills in term infants and even more in preterm ones, given their greater immaturity and risky condition. Therefore, its careful assessment at the discharge of NICUs is strongly suggested. Respiratory monitoring during feeding is quite thorny because of the high response time required to the sensors (breathing events are faster during NS), and because of the movement artifacts. Moreover, it is essential, particularly in premature infants, that any respiratory measurement be acceptable to the subject without imposing additional stress. The main techniques to record respiratory events in a clinical environment during feeding, were critically compared and analyzed in [62], even taking into consideration the above-mentioned issues. The use of a PT in a nasal cannula just inside the nostrils, as shown in Figure 9b, and of an abdominal PT (Figure 9a), can be considered suitable for clinical monitoring of respiratory events, because of their minimal invasiveness. The latter can be considered preferable in NICU where the subject may also receive oxygen via a nasal cannula, so any measure relying on nasal airflow would result useless. Quantitative information can be obtained using a nasal thermistor, embedded into a tube where the flow stream has to be canalized. However, it does not provide information about the airflow direction and it may also impose additional stress especially to a hypersensitive premature infant. A preferable solution can be the use of a pneumotachograph connected to a PT placed in a miniaturized cannula to be inserted in a nostril: it can measure both airflow and direction. However, as already said, these sensing solutions (measuring nasal airflow) may turn out to be impracticable in NICU applications. Considering a domestic post-discharge application requiring high easiness to use and portability, none of the respiratory monitoring solutions, reported in Section 3, would be easily practicable: none of them is embedded in the feeding apparatus, but they all imply the use of an additional dedicated apparatus. In a post-discharge setting, this may stress the user that is generally less inclined than a clinician to the application of any additional element on the infant's body. The same applies to the swallowing measuring systems described, as all of them imply the use of additional sensing tools. The use of PTs connected to the pharynx by means of a trans-nasal catheter has been often adopted, but it can be easily set up only in NIUCs, because it is highly invasive. A less invasive sensing solution for swallowing monitoring is represented by the use of a microphone or a pressure drum placed on the infant's neck.

The nutrient consumption is mainly recorded at the beginning and at the end of feeding (or at specific time intervals), by weighing the bottle or verifying the level of the nutrient on the graduated reservoir. Even if such methods are quite simple and not invasive, their main drawback is that they enable a global estimation of the ingested volume, but they do not allow for continuous monitoring of milk volume intake. They exclude the energetic analysis of sucking process. Moreover, as the rate of milk flow during bottle feeding plays a crucial role in feeding-related ventilator changes of both term and preterm infants [34], its assessment through reliable measures appears to be important both for infants' clinical evaluation and post-discharge monitoring. To this aim, the use of air-flow sensors (thermistors or pneumotachometers) mounted on the top of the inverted nutrient reservoir, may represent a practicable sensing solution to be further investigated to increase the level of integration and develop a portable feeding tool (it also implies the resolution of the vacuum build-up problem). The solutions using PTs to measure nutrient consumption may also be easily adopted at home for a remote continuous monitoring of infant's development, but further research is suggested for their validation in field [23,56]. The same portability can be obtained adopting an ultrasonic flow sensor, as in [55], but it represents an expensive solution. As described in Section 3, several systems adopted a calibration procedure in order to obtain a linear relation between the suction pressure and the consequent flow. However, they imply some particular expedients to eliminate hydrostatic pressure and vacuum in the nutrient reservoir, in order to make the flow solely depending on suction (see configurations in Figure 3). This affects the easiness to use and portability of the system, so it does not represent a recommended solution when both sucking components (S/E) need to be monitored, as already discussed. On the contrary, an interesting approach is the one based on the estimation of the net pressure causing the nutrient release, by measuring the pressure gradient causing the flow through a calibrated teat. Two PTs easily embeddable into the feeding teat can be used for such aim (see Figure 6) allowing both measures of sucking pressures and estimation of milk flow. Such a sensing solution allows a calibration that does not require the absence of hydrostatic pressure or vacuum in the bottle. Moreover, it is not dependent on the nutrient viscosity, which can vary among commercial formulas and breast milk, as the flow rate through an orifice is known to be independent from fluid viscosity.

## 5. Conclusions

The acquisition of efficient nutritive sucking skills is a fundamental and challenging milestone for every newborn, and even more for premature ones, as it requires the complex coordination of sucking, swallowing and breathing processes, which is usually not yet developed in premature infants at birth. Such skills and their development, if monitored, may provide objective parameters for the assessment of infants' well-being and health status, and allow predictions about later neurodevelopmental outcomes. A specifically designed tool to assess infant's oral feeding ability may provide clinicians with new devices for prognosis, diagnosis and routine clinical monitoring of newborn patients. However, such a standardized instrumental tool does not exist yet, and clinical evaluation of feeding ability is not carried out objectively, running the risk of ignoring poor feeding skills for too long. This work carries out a critical analysis of the main instrumental solutions adopted up to now for infant NS monitoring. The first step was to identify the main application fields where the objective NS assessment may contribute to improve the level of healthcare assistance: preterm assessment, term and full term assessment, and early diagnosis of later neurological dysfunctions. Different guidelines may be useful in the design and development of a measurement tool suitable for the two principal environments where it will be used: a clinical setting (the NICU in particular), and a domestic environment for post-discharge monitoring. In the latter, an instrument for monitoring NS and its development should meet two main functional requirements, besides offering reliable and valid measures: its portability and its easiness of use. In addition, non-invasiveness is strongly required in a post-discharge environment, and always preferable when dealing with preterm infants as well.

As previously discussed, some sensing solutions proposed for sucking monitoring, meeting these requirements, are based on the use of common PTs and thin catheters in a standard nipple. Even to estimate nutrient consumption, sensing solutions adopting PTs seemed to be suitable, because of their simplicity. They deserve further investigation for their reliability in field to be demonstrated. Further interest should also be addressed to the nutrient consumption estimation by means of air-flow sensors, as they show the advantages previously discussed. There is a need for sensitive, quantitative, and efficient analyses of sucking skills, first of all among preterm infants in the NICU [19]. The analytical tools for suck assessment in most NICUs are based on subjective judgment. There are obvious limitations to this approach, including reliability within and between examiners and an inability to access the fine structure of pressure dynamics, variability of suck patterning, and developmental progression [12]. The requirements for the application in clinical settings do not strictly include the portability. However, the need for a univocal assessing instrument should promote the development of the cited sensing solutions even for a clinical application, provided that reliability and validity of the measuring instrument are guaranteed. Further research should focus on the integration of the proper set of sensors for sucking monitoring on a practical feeding apparatus, and on its validation even in the case of untrained users.

Concerning the monitoring of swallowing and breathing processes during NS, some of the sensing solutions, among the ones described in literature, resulted applicable for clinical monitoring. However, none of them seemed to be easily embeddable on a feeding tool for a practical and easy use in a domestic setting, where the user is more demanding. This suggests to orient further research efforts to the design of sensing solutions for breathing and swallowing recording that can be embeddable on a simple feeding apparatus, or to the analysis of the domestic practicability of some of the less invasive solutions proposed.

Challenges and limitations discussed in this review should warrant further studies to overcome them, in order to obtain a valid and objective tool for standardizing infants' oral feeding assessment. The use of standard pre-discharge assessment devices may foster the establishment of common quantitative criteria useful to assist clinicians in planning clinical interventions. Such devices, or a simplified version of them, might be adopted also for patients' follow-up, as remote monitoring of infants at home after discharge, as everyday feeding problems can be an early symptom of disability. Besides the instrumental solution, the standardization of infants' oral feeding assessment will require a considerable work to collect the amount of data necessary to define normative indices. Moreover, the interpretation of this huge amount of data, will require further research to develop ad-hoc algorithms for data analysis.

## Figures and Tables

**Table 1.** Overview of the most significant indices adopted for the instrumental assessment of infants' NS behavior during bottle feeding.

**Application**	**Significant Indices**			

	***Sucking***	***Swallowing/Breathing***	***Consumption***	***Ref.***
Instrumental assessment of the oral feeding pattern maturation in preterm infants	Sk frequency Sucks per burst Percent sucks in burst COV_Sk_ Burst duration Inter-bursts width Inter-suck width 1st burst number of sucks IP amplitude EP amplitude S:E ratio S-E interval	COV_Sw_ COV_B_ Percentage of Apneic Swallows SW-B interface	Efficiency Nutrient intake rate Bolus size	[25,27–36]

Instrumental assessment of the oral feeding pattern maturation in term infants	Sk frequency Sucks per burst Percent sucks in burst COVsk Burst duration Inter-bursts width Inter-suck width Inconsistency Intensity	Percentage of Apneic Swallows SW-B interface Sk:Sw:B ratio Sk-B interface	Efficiency Nutrient intake rate Bolus size	[37–43]

Quantitative assessment of the oral feeding pattern for prediction of later neurodevelopmental outcomes	Sucks per burst IP amplitude EP amplitude S/E rhythmicity		Nutrient intake rate	[13,14,36,44]

**Table 2.** Overview of the physical quantities measured to monitor the NS process.

**Physical Quantities Measured**			

Sucking	Swallowing	Breathing	Nutrient Consumption
IP: [25,27,37–39,41]	Pharyngeal pressure: [25,30,33,40]	Nasal airflow/Thoracic movements: [25,30]	Total transferred nutrient: [25,28,35,40,41]

EP/IP: [14,28,29,32,35]	Hyoid bone movements: [29,32]	Thoracic movements: [29,32,33,41]	Minute transferred volume: [32,36,39]

Intranipple pressure: [30,40]	Swallows sounds: [41]		Transferred milk weight: [29]

Chin movements: [31]			

Throat-eye (S) and jaw-eye (E) distance: [44]			

**Table 3.** Overview of the measuring systems used to monitor sucking process: measurands, sensors and measurement procedures.

	**Measurand**	**Measuring Transducer**	**Measurement Procedure**	**Ref.**
Suction	Intraoral Pressure	PT	PT embedded into a catheter and placed at the tip of the nipple	[28,29,32,34,48]

			PT connected to a catheter, whose opposite tip ends into the oral cavity lumen	[10,14,16,25,35, 41,44,49–51]

			PT placed between the nipple and a flow limiting device (restriction orifice or a capillary tube)	[37,39]

	Throat movements	Videocamera and markers (DLT)	Digital video camera at 1 m from the infant's face; markers placed on the lateral angle of the eye and on the throat	[44]

Expression	Expression Pressure	PT	PT connected to a polyethylene catheter, connected to a catheter of compressible silicone rubber, placed on the nipple	[27,29,32,34,48]

			PT connected to the lumen of the nipple by means of a silicone catheter; one-way valve placed between the nipple chamber and the nutrient reservoir.	[14,35,49]

	Jaw movements	Videocamera and markers (DLT)	Digital video camera at 1 m from the infant's face; markers placed on the lateral angle of the eye and on the tip of the jaw.	[44]

		Strain gauge Transducer	Strain gauge transducer attached between the infant's forehead and the chin	[10]

Sucking events (no S/E distinction)	Intranipple pressure	PT	PT connected to the lumen of the nipple by means of a silicone catheter	[30,33,40,51,52]
	Chin movements	Strain-gauge	Stretch-sensitive strain gauge placed under the infant's chin and secured over the zygomatic bones; its wire connector inserted into a plethysmograph	[31,53]

**Table 4.** Overview of the measuring systems used to monitor swallowing and breathing process: measurands, sensors and measurement procedures.

	**Measurand**	**Measuring** **Transducer**	**Measurement Procedure**	**Ref.**
Swallow events	Pharyngeal pressure	PT	PT connected to a catheter introduced transnasally with the tip at the oropharynx	[25,30,33,40,50,52]

	Hyoid bone movements	PT	PT connected to a small drum placed onto the hyoid region of the infant' s neck	[29,32,48]

	Swallow sounds	Microphone	Miniature microphone attached to the infant's throat	[10,41,49]

Breathing flow	Nasal air-flow	Thermistor/Termocouples	Sensor placed below the nostrils	[10,25,30, 40,49]

		Pneumotachograph and PT	Sensor placed in a nostril	[43]

		PT	PT connected to a catheter inserted just into the nares (information about direction only)	[10]

Breathing movements	Thoracic movements	Strain-gauge	Respiratory band around the infant's chest	[25,30,33,41,50]

		PT	Pressure drum placed below the chest	[29,32]

**Table 5.** Overview of the measuring systems used to monitor the nutrient consumption: measurands, sensors and measurement procedures.

	**Measurand**	**Measuring Transducer**	**Measurement Procedure**	**Ref.**
Nutrient flow	Air flow into the bottle	Thermistor	Sensor fixed to the lumen of a rigid tube inserted into the reservoir bung	[10]

		Pneumotachometer	Inserted at the top of the nutrient reservoir	[54]

	Milk flow	Ultrasonic flow transducer	Placed between the nipple and the bottle	[55]

Nutrient volume	Nutrient weight	Balance	Residual milk weighted continuously or at intervals	[29,48]

	Nutrient volume	Graduated reservoir	Residual milk volume measured from the graduated reservoir	[27,32,39]

	Hydrostatic Pressure	PT	PT connected to a catheter placed at the base of the residual liquid column;	[50,56]

	Intra-bottle vacuum	PT	PT connected to the top of the bottle, where the vacuum creates	[23]
